# Being a *Participant* Matters: Event-Related Potentials Show That Markedness Modulates Person Agreement in Spanish

**DOI:** 10.3389/fpsyg.2019.00746

**Published:** 2019-04-24

**Authors:** José Alemán Bañón, Jason Rothman

**Affiliations:** ^1^Centre for Research on Bilingualism, Department of Swedish and Multilingualism, Stockholm University, Stockholm, Sweden; ^2^Department of Languages and Linguistics, Faculty of Humanities, Social Sciences, and Education, The Arctic University of Norway, Tromsø, Norway; ^3^Centro de Ciencia Cognitiva, Universidad Nebrija, Madrid, Spain

**Keywords:** ERP, P600, late anterior negativity, markedness, person agreement, prediction, Spanish

## Abstract

The present study uses event-related potentials to examine subject–verb person agreement in Spanish, with a focus on how markedness with respect to the speech participant status of the subject modulates processing. Morphological theory proposes a markedness distinction between first and second person, on the one hand, and third person on the other. The claim is that both the first and second persons are *participants* in the speech act, since they play the *speaker* and *addressee* roles, respectively. In contrast, third person refers to whomever is neither the *speaker* nor the *addressee* (i.e., it is unmarked for person). We manipulated speech participant by probing person agreement with both first-person singular subjects (e.g., *yo…lloro* “I…cry-_1ST PERSON-SG_”) and third-person singular ones (e.g., *la viuda…llora* “the widow…cry-_3RD PERSON-SG_”). We also manipulated agreement by crossing first-person singular subjects with third-person singular verbs (e.g., *yo…^∗^llora* “I…cry-_3RD PERSON-SG_”) and vice versa (e.g., *la viuda…^∗^lloro* “the widow…cry-_1ST PERSON-SG_”). Results from 28 native speakers of Spanish revealed robust positivities for both types of person violations, relative to their grammatical counterparts between 500 and 1000 ms, an effect that shows a central-posterior distribution, with a right hemisphere bias. This positivity is consistent with the P600, a component associated with a number of morphosyntactic operations (and reanalysis processes more generally). No negativities emerged before the P600 (between 250 and 450 ms), although both error types yielded an anterior negativity in the P600 time window, an effect that has been argued to reflect the memory costs associated with keeping the errors in working memory to provide a sentence-final judgment. Crucially, person violations with a marked subject (e.g., *yo…^∗^llora* “I…cry-_3RD PERSON-SG_”) yielded a larger P600 than the opposite error type between 700 and 900 ms. This effect is consistent with the possibility that, upon encountering a subject with marked features, feature activation allows the parser to generate a stronger prediction regarding the upcoming verb. The larger P600 for person violations with a marked subject might index the reanalysis process that the parser initiates when there is a conflict between a highly expected verbal form (i.e., more so than in the conditions with an unmarked subject) and the form that is actually encountered.

## Introduction

The present study uses event-related potentials (ERPs) to investigate the processing of subject–verb person agreement in Spanish. An example of how person information is encoded in the Spanish verb is provided in (1). As can be seen, the form of the verb *entrenar* “to train,” which is inflected in the simple present for singular subjects, varies systematically depending on whether the subject is the speaker (*yo*, first-person singular), the addressee (*tú*, second-person singular), or someone else (*el atleta* “the athlete”).

**Table:** 

(1)	a.	Yo	entren**o**.	
		I	train-_1ST PERSON-SG_	
	b.	Tú	entren**as**.	
		You-_SG_	train-_2ND PERSON-SG_	
	c.	El	atleta	entren**a**.
		The	athlete	train-_3RD PERSON-SG_

A number of theoretical proposals have drawn a distinction between first and second person on the one hand, and third person, on the other (e.g., Jakobson, 1971; Harris, 1996; Harley and Ritter, 2002; McGinnis, 2005; Bianchi, 2006). The idea is that both first and second person are *participants* in the speech act, since they play the *speaker* and *addressee* roles, respectively. Third person, in contrast, is not a speech participant and merely refers to someone who is neither the speaker nor the addressee. This distinction bears directly upon the concept of markedness, the observation that different feature values carry differential weight (e.g., Battistella, 1990; Bonet, 1995; Corbett, 2000; Cowper, 2005). The claim is that third person, not being a speech participant, is unmarked relative to first and second person (e.g., Harley and Ritter, 2002; Bianchi, 2006; Wechsler, 2011). Our study investigates if and how markedness with respect to the speech participant status of the subject modulates person agreement resolution online. We do this by comparing sentences with a first-person singular subject (speaker role) to sentences with a third-person singular subject (default person).

An influential proposal formalizing this markedness distinction between first/second and third person is Harley and Ritter (2002). Harley and Ritter (2002) offer a feature geometry analysis for person (and number) where features, such as *participant*, are privative rather than binary. For the person feature, this means that only first and second person have the status of true grammatical persons. In contrast, third person carries no person specification at all (see also Benveniste, 1971; Kayne, 2000; McGinnis, 2005; Adger and Harbour, 2006; Wechsler, 2011). Contrastive proposals treat third person as a true grammatical person, one that is specified as “non-participant.” This is, for example, what Nevins (2007, 2011) argues for third-person pronouns (but not for lexical determiner phrases “DP,” which he assumes carry no person specification). Crucially, despite these differences with respect to third-person pronouns, there is consensus that only the first and second persons are participants in the speech act. In fact, Bianchi (2006, p. 2026) suggests that this distinction might be universal.

This conceptual distinction between first/second and third person is consistent with typological data showing (a) that third person often distributes differently from first and second person crosslinguistically and (b) that third person is morphologically unmarked. For example, Forchheimer (1953) points out that some languages have specific pronouns for the first and second persons, but not the third (i.e., demonstratives are used instead, as in Halh Mongolian or Telugu; see Harley and Ritter, 2002). In addition, in some languages, first and second person show overt agreement, but third person does not. This is indeed what Harris (1996) argues for Spanish (i.e., that there is only first- and second-person verbal morphology). Finally, third-person pronouns are more likely to show gender distinctions than first- or second-person pronouns. Since the third person is not a speech participant, its referent in the speech act is independent from the discourse and, thus, more likely to show distinctions that are also independent from the discourse, such as gender^1^. We see this in Spanish, where gender distinctions only emerge in the third-person pronoun^2^.

**Table:** 

(2)	a.	yo
		_1ST PERSON-SG_
	b.	tú
		_2ND PERSON-SG_
	c.	él/ella/ello
		_3RD PERSON-SG-MASC/FEM/NEUT_

An interesting question that arises is whether these markedness distinctions impact the establishment of person dependencies online. In the psycholinguistic literature, a self-paced reading study by Carminati (2005) provides psycholinguistic validity for the differential treatment of first and second person on the one hand, and third person on the other. Carminati examined bi-clausal sentences in Italian where she manipulated the type of cue that served to disambiguate a null pronoun toward its antecedent (e.g., *Quando Maria ha litigato con me, ero…* “when Maria quarreled with me,” *pro* was-_1ST PERSON-SG_). The logic behind this paradigm is that, in Italian, null pronouns show a strong preference toward the subject position (i.e., *Maria*). Carminati found that having to establish co-reference between a null pronoun and a non-preferred antecedent (i.e., the object, the underlined first-person pronoun *me*) carried a smaller penalty (in terms of reaction time) when the disambiguating verb was inflected for first or second person, relative to third person (e.g., *Quando ho litigato con Maria, era…* “when quarreled-_1ST PERSON-SG_ with Maria,” *pro* was-_3RD PERSON-SG_). In contrast, no differences emerged between the first and second persons. This suggests that first- and second-person cues are stronger than third-person cues, consistent with the possibility that they carry greater cognitive weight.

Outside the domain of person agreement, the literature on *agreement attraction* has provided additional evidence for the psycholinguistic validity of markedness, in this case for number and, to a lesser extent, gender (attraction is argued not to be possible for person; e.g., Den Dikken, 2011; Nevins, 2011). In attraction, a finite verb agrees in number with a noun other than its controller subject, one that is structurally inaccessible, as in *The key to the cabinets^∗^are…* (production: Bock and Miller, 1991; Antón-Méndez et al., 2002; comprehension: Pearlmutter et al., 1999; Wagers et al., 2009; Dillon et al., 2013; Acuña Fariña et al., 2014; Lago et al., 2015). Importantly, attraction tends to occur when the attractor noun (i.e., *cabinets*) is plural (i.e., marked for number). Singular nouns (i.e., unmarked for number) rarely attract. Thus, both Carminati’s study (2005) and the literature on attraction provide interesting evidence that markedness impacts the processing of person and number dependencies, at least in contexts that involve more than one trigger noun (whether or not they are licensed as controllers). In the present study, we examine whether markedness differences with respect to the speech participant status of the subject (speaker vs. default person) modulate person agreement in simpler sentences with an unambiguous subject.

One possibility is that the marked status of the subject will allow the parser to compute agreement as a top-down mechanism (e.g., Nevins et al., 2007; Wagers and Phillips, 2014). A number of proposals assume that agreement is a predictive procedure (e.g., Gibson, 1998, 2000; Wagers et al., 2009; Dillon et al., 2013; Lago et al., 2015; but see for example, Nicol et al., 1997; Pearlmutter et al., 1999), but little is known as to the role of markedness in predictive processing. Nevins et al. (2007) proposed that, for subject–verb agreement, feature activation at the subject might allow the parser to generate a stronger prediction regarding the form of the upcoming verb (Wagers and McElree, unpublished also posit that the parser can conclude more from the presence than the absence of a feature). This is a possibility that we evaluate in the present study. Herein, we use ERPs, brain responses which are time-locked to stimuli of interest and which provide high temporal resolution.

## ERP Literature on Agreement

The ERP literature on agreement (as a general phenomenon) has mainly focused on the P600, a positive-going wave that typically emerges between 500 and 900 ms in central-posterior electrodes (see Molinaro et al., 2011a for a review). The functional significance of the P600 is still debated. It was initially interpreted as an index of difficulty at the level of the syntax (reanalysis, repair, integration), as it was found for morphosyntactic anomalies (e.g., Hagoort et al., 1993; Osterhout and Mobley, 1995; Friederici et al., 1996), garden-path sentences (e.g., Osterhout and Holcomb, 1992), and grammatical but complex sentences that require the integration of displaced elements (e.g., Kaan et al., 2000). Some have also argued that the P600 encompasses two separate phases, which are sensitive to different factors and show different topography (e.g., Hagoort and Brown, 2000). This proposal has received interest in the agreement literature, where the late phase of the P600 (∼700–900 ms, argued to be sensitive to repair mechanisms) has been found to be modulated by feature distinctions. For example, Barber and Carreiras (2005) found it to be larger for gender than number in Spanish, and Mancini et al. (2011a) found it to be larger for person than number in Spanish (but see Alemán Bañón et al., 2012; Chow et al., 2018a).

The finding that certain types of semantic anomalies (e.g., Kolk et al., 2003; Kuperberg et al., 2003, 2006; Kim and Osterhout, 2005) and non-linguistic stimuli (Patel et al., 1998) sometimes also yield a P600 has prompted alternative proposals where the P600 is viewed as an index of reanalysis in general, as opposed to core morphosyntactic processing (see Tanner et al., 2017). For example, van de Meerendonk et al. (2010) argue that the P600 reflects the reanalysis process triggered by a strong conflict between a highly expected linguistic element (e.g., a word, a morpheme) and the encountered input, thus assuming that the P600 is sensitive to the violation of top–down expectations. Other proposals argue that the P600 reflects (non-exclusively morphosyntactic) combinatorial processing (Kuperberg, 2007) or well-formedness checking (e.g., Bornkessel-Schlesewsky and Schlesewsky, 2008). We do not elaborate on these proposals here, since the purpose of our study is not to tease them apart (see also Brouwer et al., 2012; Van Petten and Luka, 2012). What is important for the purposes of the present study is that the P600 consistently emerges for agreement errors across languages, agreement types (e.g., person, number, gender), and syntactic contexts (e.g., subject–verb, determiner–noun, noun–adjective, etc.) (see Table 1 in Molinaro et al., 2011a, p. 910).

The same is not true of a negativity that sometimes precedes the P600 between ∼300 and 500 ms. In some studies, this negativity shows an anterior distribution, sometimes with a left hemisphere bias. In others, it is more broadly distributed, spanning over central-posterior areas. This topographical variability has generated much debate regarding the identity of this component. Some refer to it as a Left Anterior Negativity (LAN), a component argued to index automatic morphosyntactic processing (e.g., Friederici et al., 1996; Friederici, 2002; De Vincenzi et al., 2003; Barber and Carreiras, 2005; Molinaro et al., 2008; Mancini et al., 2011a; Caffarra and Barber, 2015) or the working memory costs associated with the processing of long-distance dependencies (e.g., Kluender and Kutas, 1993; Fiebach et al., 2002; see a review in Molinaro et al., 2011a). In the agreement literature, it has been argued that the Left Anterior Negativity is more likely to emerge when the dependency is local (e.g., determiner–noun), the agreement cues are overt, and the reference site is hemisphere-neutral (e.g., Molinaro et al., 2011a,b).

Other researchers have argued that the LAN is reminiscent of the N400 (e.g., Service et al., 2007; Guajardo and Wicha, 2014; Tanner and van Hell, 2014; but see Molinaro et al., 2015), a component related to lexical retrieval and semantic integration (see Lau et al., 2008 for a review). Recent work by Caffarra et al. (2019), however, suggests that the LAN can characterize agreement progressing independently of the N400 (at least, for determiner–noun gender errors in Spanish). Yet, others have argued that agreement violations yield either a LAN or an N400, depending on the levels of representation (e.g., morphosyntax, discourse) that are disrupted by the error (e.g., Mancini et al., 2011a). Importantly, in many studies on agreement, this negativity is simply absent (e.g., Nevins et al., 2007; Frenck-Mestre et al., 2008; Hammer et al., 2008), even for local agreement errors in languages with rich morphosyntax (e.g., Wicha et al., 2004; Alemán Bañón et al., 2012, 2014). Herein, we will focus mainly on the P600, which is the most consistent ERP signature of agreement, although we will also investigate the LAN. In the next section, we review how these components have informed our understanding of how person dependencies are established in real-time comprehension.

## ERP Literature on Person Agreement

A number of studies have used ERP to investigate agreement, but only a few have manipulated person dependencies. Silva-Pereyra and Carreiras (2007) found robust P600 effects for single person violations in Spanish between 700 and 900 ms (e.g., *yoentiendo/^∗^entiendes* “I-_1ST PERSON-SG_ understand-_1ST PERSON-SG_/^∗^understand-_2ND PERSON-SG_). This positivity emerged earlier (500–700 ms) for combined person + number violations. In addition, only combined violations showed an anterior negativity (300–450 ms), which was not left-lateralized. Rossi et al. (2005) also reported this biphasic pattern (LAN-P600) for single person violations in German, although both components emerged later in Rossi et al.’s study.

Nevins et al. (2007) examined subject–verb agreement in Hindi with a design that includes both single (number, gender) and combined errors (number + gender, person + gender). Crucially for the purposes of the present study, they examined whether agreement is computed as a bottom-up or top-down (i.e., predictive) mechanism. In the latter case, Nevins et al. hypothesized that combined violations would yield a larger P600 than single errors, since the distance between the predicted and encountered forms increases as a function of the number of features violated. Their results showed equally robust P600 effects for single number, single gender, and combined number + gender violations (not preceded by a LAN). Combined person + gender errors yielded an earlier and larger P600 than all other error types, but a follow-up study suggested that this was due to person being orthographically more marked/salient in the Devanagari script. Thus, these results are inconclusive as to whether agreement checking takes place top-down. However, Nevins et al. suggest that this might have been due to their using subjects with a default status (i.e., third person, singular, masculine), which might have failed to activate the relevant features. We address this question in our study, by specifically manipulating the markedness of the subject with respect to the person feature (first vs. third person).

In another study looking at Spanish, Mancini et al. (2011a) found that person violations (e.g., *el cocinero^∗^cocinaste…* “the cook-_3RD PERSON-SG_ cooked-_2ND PERSON-SG_”) yielded an N400-P600 biphasic pattern, relative to control sentences (e.g., *los cocineroscocinaron…* “the cook-_3RD PERSON-PL_ cooked-_3RD PERSON-PL_”), whereas number violations (e.g., *el cocinero^∗^cocinaron…* “the cook-_3RD PERSON-SG_ cooked-_3RD PERSON-PL_”) elicited a LAN-P600 biphasic pattern. In addition, the early phase of the P600 (500–800 ms) was broader, and the late phase (800–1000 ms) larger, for person relative to number errors. The authors argue that the qualitative differences between person (N400) and number (LAN) reflect the different interpretative procedures associated with each feature. Their claim is that only person violations disrupt the process of building a discourse representation, since the parser cannot assign a speech role (speaker, addressee) to the subject (see Tanner and van Hell, 2014 for an alternative proposal regarding N400 effects for agreement errors).

These qualitative differences between person and number were not replicated by Zawiszewski et al. (2016). The authors compared the effects of person, number, and person + number violations in Basque (e.g., *zuk…utzi duzu/^∗^dut/^∗^duzue/^∗^dugu* “you-_2ND PERSON-SG_ left have-_2ND PERSON-SG_/^∗^left have-_1ST PERSON-SG_/^∗^left have-_2ND PERSON_ _-PL_/^∗^left have-_1ST PERSON-PL_) and found an N400-P600 biphasic pattern (and a late frontal negativity) for all error types. Interestingly, the P600 was larger in the two conditions with a person mismatch, which the authors interpret as evidence that person is more salient than number, although they cannot rule out that this was due to orthographic differences between the critical words (e.g., Nevins et al., 2007). The N400 effect for person (and number) violations is accounted for by the fact that the Basque verb also instantiates object agreement, which requires the parser to check thematic relations (upon encountering a disagreeing verb).

To our knowledge, the only study that has manipulated markedness in an examination of person agreement is Mancini et al. (2018). The authors probed two types of person dependencies in Basque that differed with respect to the speech participant status of the subject (first-person plural: marked vs. third-person plural: unmarked). Their design encompassed errors where a first-person plural subject mismatched a third-person plural verb (*japoniarr-ok…ikasi dugu/^∗^dute* “Japanese-_1ST PERSON-PL_ learned have-_1ST PERSON-PL_/^∗^learned have-_3RD PERSON-PL_”) and errors where a third-person plural subject mismatched a first-person plural verb (*japoniarr-ek…ikasi dute/^∗^dugu* “Japanese-_3RD PERSON-PL_ learned have-_3RD PERSON-PL_/^∗^learned have-_1ST PERSON-PL_”). The authors hypothesized that the latter error type would yield a qualitatively different P600, because the marked person features of the verb (first-person) could extend to the unmarked subject (third-person) and “rescue” the violation. In fact, such a mismatch is ungrammatical in Basque, but not in languages like Bulgarian, Modern Greek, Swahili, or Spanish (example from Spanish: *los investigadores somos tenaces* “the researchers-_3RD PERSON-PL_ are-_1ST PERSON-PL_ tenacious”), a phenomenon known as *unagreement* (e.g., Hurtado, 1985; Höhn, 2016). Both error types yielded an N400, but only “first-person plural subject + third-person plural verb” errors showed a P600. The authors argue in favor of their hypothesis, although they cannot rule out the possibility that participants treated violations on first-person plural verbs as grammatical unagreement (they accepted them at a rate of 42% in the judgment task, and ERPs were calculated without excluding incorrectly judged trials), especially as they were highly proficient bilingual speakers of Spanish. This would be consistent with Torrego and Laka’s claim (2015) that unagreement is grammatical in Basque, although it is subject to individual differences. Importantly, previous work by Mancini et al. (2011b) showed a qualitatively similar processing profile (N400, no P600) for unagreement sentences in Spanish. Thus, although Mancini et al.’s results (2018) are interesting, the evidence that outright violations with unmarked subjects are salvageable requires further exploration (see Mancini et al., 2018 for counterarguments).

Importantly, Mancini et al.’s results (2018) show that markedness does modulate person agreement. Whether “third-person plural subject + first-person plural verb” combinations yielded no P600 effect because (1) the unmarked status of the subject makes an outright person violation less disruptive (potentially due to the participants’ bilingualism with Spanish, a language that clearly allows this) or (2) because the Basque grammar itself simply allows it (e.g., Torrego and Laka, 2015), what is important is that the speech participant status of the subject affects person agreement resolution. Thus, Mancini et al.’s study (2018) adds to a small ERP literature showing that markedness modulates agreement processing (e.g., Deutsch and Bentin, 2001; Kaan, 2002). Outside the realm of person agreement, a previous study from our own lab (Alemán Bañón and Rothman, 2016) was the first to investigate how markedness affects the processing of noun–adjective number and gender agreement (in Spanish). In that study, we examined markedness by manipulating the number/gender of the trigger nouns and their agreeing adjectives (e.g., *una catedral que parecía inmensa* “a cathedral-_FEM-SG_ that looked huge-_FEM-SG_”). Following Nevins et al. (2007), one of our hypotheses was that the parser might be more likely to engage in predictive processing when the controller noun carried marked features (gender: feminine; number: plural), due to feature activation. In that case, our prediction was that errors of the kind “marked noun + unmarked adjective” might result in a larger P600 than the opposite error type, given that a prediction would be generated but unmet. Instead, we found that violations realized on marked adjectives (the opposite error type) yielded an earlier P600 for both number and gender. In addition, the P600 was larger for number errors realized on plural adjectives (e.g., Deutsch and Bentin, 2001; Kaan, 2002). Although our results provide evidence that markedness modulates agreement, they do not provide evidence that markedness triggers predictive processing. One possibility, however, is that the syntactic frame where we examined agreement was not sufficiently constraining to allow for the generation of strong predictions. That is, although an adjective carrying agreement features was likely to appear after the structure “Noun that looked/seemed…,” other continuations were possible (e.g., *una catedral que parecía desafiar la gravedad* “a cathedral-_FEM-SG_ that seemed to defy gravity”). However, the same is not true of subject–verb agreement, where the presence of a subject allows for the strong prediction that a verb will appear further down the line. We address this question in the present study.

## The Present Study: Research Questions and Predictions

The present study examines the processing of two types of person dependencies in Spanish. Crucially, the study is among the first to investigate how the online resolution of person agreement is impacted by markedness. Samples of the structure where we manipulated markedness (and agreement) can be seen in (3-6). The agreement relation of interest is that between the subject and the verb (underlined). Our design examines markedness by manipulating the speech participant status of the subject, such that half of the sentences had a first-person subject (marked for person: speaker role; see 3 and 4) and the other half, a third-person subject (unmarked for person; see 5 and 6). Agreement was manipulated by crossing each subject type with a verb showing the opposite person inflection. Unlike Mancini et al. (2018), we only used singular subjects and, thus, both types of person violations had an unambiguously ungrammatical status in Spanish (i.e., singular unagreement is not licensed in Spanish; see Torrego, 1996).

**Table:** 

(3)	Yo	a menudo acaricio a	los caballos.
	I-_1ST PERSON-SG_	often pet-_1ST PERSON-SG CASE_	the horses
(4)	Yo	a menudo acelero	en la autopista.
	I-_1ST PERSON-SG_	often speed up-_1ST PERSON-SG_	on the highway.
(5)	El cartero	a menudo acaricia a	los gatos.
	the postman-_3RD PERSON-SG_	often pet-_3RD PERSON-SG CASE_	the cats
(6)	El conductor	a menudo acelera	en la carretera.
	the driver-_3RD PERSON-SG_	often speed up-_3RD PERSON-SG_	on the road.

As a first step, we will examine which ERP components are associated with violations of person agreement. Based on the previous literature, our prediction is that both types of person violations will yield a P600, which is a reliable finding across studies (Nevins et al., 2007; Silva-Pereyra and Carreiras, 2007; Zawiszewski et al., 2016; Mancini et al., 2011a, 2018). Predictions regarding negative effects (LAN, N400) preceding the P600 are less straightforward, since these effects only emerged in the studies by Mancini et al. (2011a, 2018) and Zawiszewski et al. (2016) (and Rossi et al., 2005 found a LAN). In addition, Zawiszewski et al. (2016) interpret the N400 as evidence that person violations compromise thematic role assignment, given that the Basque verb also instantiates object agreement, an operation that does not apply to Spanish.

Our main research question concerns how markedness will impact person agreement resolution. We evaluate two possible scenarios. First, “third-person subject + first-person verb” violations could yield an earlier and larger P600 relative to “first-person subject + third-person verb errors.” This is because first-person verbs are marked relative to third-person ones (e.g., Harris, 1996). This would be consistent with what we found in Alemán Bañón and Rothman (2016) and would constitute further evidence that the parser can more easily detect violations realized on marked elements or that these are more disruptive (e.g., Friederici et al., 2001; Kaan, 2002; Nevins et al., 2007). Alternatively, if Nevins et al.’s (2007) proposal that the parser is more likely to engage in predictive processing when the subject carries marked features is on the right track, it is possible that violations of the type “first-person subject + third-person verb” (hereinafter “marked subject violations”) will yield a larger P600 than “third-person subject + first-person verb” errors (hereinafter “unmarked subject violations”). It is also possible that the positivity will span over frontal areas, given recent proposals linking frontal positivities to prediction disconfirmation (e.g., DeLong et al., 2011; see Van Petten and Luka, 2012 for a review). This is because the marked status of the first-person subject (i.e., speaker) would activate the person feature, allowing the parser to generate a prediction regarding the specification of the upcoming verb. The same is not true of lexical subjects such as *el conductor* “the driver,” which do not carry a person feature (e.g., Bianchi, 2006) ^3^. To sum up, Alemán Bañón and Rothman’s (2016) proposal predicts that the verb’s markedness (as in 7) will impact processing at the violating verb, whereas Nevins et al.’s proposal predicts that it is the subject’s markedness (as in 8) that will impact processing at the verb.

**Table:** 

(7)	la viuda	^∗^lloro
	the widow-_UNMARKED_	cry-_MARKED_
(8)	yo	^∗^llora
	I-_MARKED_	cry-_UNMARKED_

## Materials and Methods

Before the testing began, the study was reviewed by the relevant research ethics committee at the University of Reading and received clearance (project number: 2014-031-JAB). All participants provided their informed written consent to take part in the study.

### Participants

The participants include 28 native speakers of Spanish (16 females; age range: 18–38; mean age: 27). Data from 27 of these participants (from a different study) were reported in Alemán Bañón and Rothman (2016). All participants indicated being right-handed, and this was confirmed via the Edinburgh Handedness Inventory (Oldfield, 1971). In addition, they all reported having no history of cognitive or neurological damage/diseases. They all spoke one or more foreign languages (mainly English) to varying levels of proficiency, and four of them identified themselves as speakers of another one of Spain’s co-official languages (Catalan, Galician) or Spanish Sign Language. They all received financial compensation for their time.

### Materials

The materials comprise 160 single-clause sentences assigned to one of the four conditions in Table 1. All sentences follow the structure: subject + temporal adverb *a menudo* “often” + verb in the simple present + continuation (i.e., direct object or prepositional phrase). Half of the sentences (see conditions 1–2 in Table 1) include a lexical DP subject (e.g., *el cazador* “the hunter”), which corresponds to the default person (third person). In the grammatical version (condition 1), the verb is in the third-person singular. In the ungrammatical version (condition 2), the verb is incorrectly inflected as first-person singular, which is marked for person. In the other 80 sentences (conditions 3–4), the subject is the first-person singular pronoun *yo* (marked person: speaker). In the correct version (condition 3), the verb carries first-person singular inflection. In the ungrammatical version (condition 4), the verb shows third-person singular features and is, therefore, incorrectly underspecified for person. We chose the first as opposed to the second person as the marked subject for two reasons. First, only the first person allowed us to match the target verbs for length (e.g., *lloro* “cry-_1ST PERSON-SG_” vs. *llora* “cry-_3RD PERSON-SG_”; compare to *lloras* “cry-_2ND PERSON-SG_”). Second, there is substantial variability with respect to the use of the second person across varieties of Spanish, even within European Spanish (e.g., Green, 1988).

**Table 1:** Sample of the materials, including the conditions examining person agreement with third-person singular subjects (grammatical, ungrammatical), the conditions examining person agreement with first-person singular subjects (grammatical, ungrammatical), and the fillers.

3^rd^ person singular subject
**Grammatical**
1. *El cazador a menudo acampa en la montaña.*
The hunter-_3RD PERSON-SG_ often camp-_3RD PERSON-SG_ in the mountain
**Unmarked-subject violation**
2. *El cazador a menudo ^∗^acampo en la montaña.*
The hunter-_ 3RD PERSON-SG_ often camp-_1ST PERSON-SG_ in the mountain

**1^st^ person singular subject**

**Grammatical**
3. *Yo a menudo canto en la ducha.*
I-_1ST PERSON-SG_ often sing-_1ST PERSON-SG_ in the shower
**Marked-subject violation**
4. *Yo a menudo ^∗^canta en la ducha.*
I-_ 1ST PERSON-SG_ often sing-_3RD PERSON-SG_ in the shower

**Fillers**

*Nosotros somos muy comprensivos y ellos tambièn.*
We-_1ST PERSON-PL_ are very understanding and they-_3RD PERSON-PL_ too
*Ellas son más puntuales que tú.*
They-_3RD PERSON-PL_ are more punctual than you-_2ND PERSON-SG_


In sum, markedness was manipulated via the speech participant status of the subject and its corresponding verb (*el cazador…caza* “the hunter-_3RD PERSON-SG_ hunt-_3RD PERSON-SG_,” *yo…cazo* “I-_1ST PERSON-SG_ hunt-_1ST PERSON-SG_”) and agreement was manipulated by pairing up first-person subjects with third-person verbs, and third-person subjects with first-person verbs. The adverb *a menudo* “often” intervened between the subject and verb in order to create some linear distance between the agreeing elements. We reasoned that this might give the parser a better opportunity to engage in predictive processing, since additional time is available for prediction generation (e.g., Chow et al., 2016, 2018b). Thus, if subject–verb agreement is ever predictive, we thought that this would be an appropriate set-up to explore such a possibility.

For the conditions with third-person subjects, we used lexical subjects (as opposed to third-person singular pronouns) for two reasons. First, it allowed us to diversify the stimuli as much as possible. Most importantly, as discussed in Section “Introduction,” there is disagreement in the literature regarding whether third-person pronouns carry any person specification (e.g., Harley and Ritter, 2002 argue that they do not; Nevins, 2007 argues the reverse). In contrast, there seems to be agreement that lexical DPs are underspecified for person (e.g., Den Dikken, 2011; Nevins, 2011). Since the same could not be done in the conditions with first-person subjects, the fillers were designed so as to mitigate the salience of the first-person singular pronoun *yo*, which participants saw in 80 sentences. Therefore, the fillers involved 40 instances of the second-person singular pronoun *tú* “you,” 40 instances of the first-person plural pronouns *nosotros/nosotras* “we-_MASC/FEM_,” and 80 instances of the third-person plural pronouns *ellos/ellas* “they-_MASC/FEM_”). All materials are provided in Supplementary File 1.

Each inflected verb (e.g., *llora* “cry-_3RD PERSON-SG_,” *lloro* “cry-_1ST PERSON-SG_”) was used twice, once with a third-person singular subject and once with a first-person singular subject (e.g., *La viuda a menudo llora/^∗^lloro en la iglesia* “the widow often cry-_3RD PERSON-SG_/^∗^cry-_1ST PERSON-SG_ in church”; *Yo a menudo lloro/^∗^llora en las películas* “I often cry-_1ST PERSON-SG_/^∗^cry-_3RD PERSON-SG_ at the movies”). This was done to ensure that all properties associated with a given verb (e.g., meaning, argument structure, lexical aspect, etc.) would be held constant across the two markedness conditions. With the exception of the subject, all sentences across the two markedness conditions were therefore identical up to the critical verb. Since the testing took place in two separate sessions, we distributed the materials in such a way that participants would only see one token of each verb per session.

Since the verbs were the same across markedness conditions, they were controlled with respect to number of characters [mean length of verbs inflected as third-person singular: 6.56; mean length of verbs inflected as first-person singular: 6.57; *t*(79) = 0.445, *p* = 0.658]. Mean length was, however, not exactly the same, due to five verbs showing certain conjugational or orthographic idiosyncrasies (e.g., *conduce* “drive-_3RD PERSON-SG_” vs. *conduzco* “drive-_1ST PERSON-SG_”; *sigue* “follow-_3RD PERSON-SG_” vs. *sigo* “follow-_1ST PERSON-SG_”). It was not possible to match the critical verbs with respect to frequency of use. We calculated the log frequency of each form with the EsPal database (Duchon et al., 2013), and found that third-person singular forms were significantly more frequent than first-person singular ones. This is unsurprising, given that default forms (i.e., third-person singular) have a wider syntactic distribution. Notice that a similar issue arose in Mancini et al.’s (2011a) study and that information about frequency is not provided in most other ERP studies on person agreement (e.g., Rossi et al., 2005; Nevins et al., 2007; Silva-Pereyra and Carreiras, 2007; Zawiszewski et al., 2016) ^4^. Finally, the position of the critical verb was always mid-sentence, and it was similar across markedness conditions (conditions 1–2: word #5; conditions 3–4: word #4).

These materials were intermixed with 240 sentences (160 ungrammatical) from a separate study that examines noun–adjective number and gender agreement, but does not manipulate subject–verb agreement (reported in Alemán Bañón and Rothman, 2016). All 80 fillers were grammatical, which brought the ratio of grammatical to ungrammatical sentences to 1/1. A sample of each filler type is provided in Table 1.

### Procedure

The testing was divided into two 3-hour sessions (e.g., O’Rourke and Van Petten, 2011; Alemán Bañón et al., 2012). Each EEG recording included 240 sentences (with an equal number of items per condition, including the fillers) and took approximately 1 h. Participants read the sentences quietly. The sentences were presented one word at a time, in random order. After each sentence, participants provided a grammaticality judgment, similar to previous ERP studies on person agreement (e.g., Rossi et al., 2005; Nevins et al., 2007; Silva-Pereyra and Carreiras, 2007; Mancini et al., 2011a, 2018; Zawiszewski et al., 2016). Participants received instructions to favor accuracy over speed while judging the sentences, to avoid blinks and muscle movements while reading them, and to rest their eyes between trials. At the beginning of each session, participants completed an eight-trial practice set (four ungrammatical) so that they would become acquainted with the task. None of the practice trials involved agreement errors or nouns/verbs from the experimental stimuli. Participants received feedback for the first three practice trials. The experiment began right after. Each session comprised six 40-sentence blocks, separated by five short breaks. Sentence presentation was carried out in *Paradigm*, by Perception Research Systems Inc. (Tagliaferri, 2005).

Each trial began with a fixation cross, which remained in the center of the screen for 500 ms. Then, the presentation of the sentence began, one word at a time, using the Rapid Serial Visual Presentation method. Each word remained on the screen for 450 ms, followed by a 300 ms pause (e.g., Alemán Bañón et al., 2012; see Molinaro et al., 2011a). Upon presentation of the last word (marked with a period), there was a 1000 ms pause. Right after, participants saw the prompts for the Grammaticality Judgment Task (GJT), the words *Bien* “good” and *Mal* “bad” for grammatical and ungrammatical sentences, respectively. The prompts remained visible until participants provided a response, which they did with their left hand (middle and index fingers, respectively). After the behavioral response, we added an inter-trial interval ranging between 500 and 1000 ms, pseudo-randomly varied at 50 ms increments.

### EEG Recording and Analysis

The EEG was recorded with the Brain Vision Recorder software (Brain Products, GmbH, Germany) from 64 sintered Ag/AgCl electrodes mounted in an elastic cap (Easycap, Brain Products, GmbH, Germany). The placement of the electrodes followed the 10% system (midline: FPz, Fz, Cz, CPz, Pz, POz, Oz; hemispheres: FP1/2, AF3/4, AF7/8, F1/2, F3/4, F5/6, F7/8, FC1/2, FC3/4, FC5/6, FT7/8, FT9/10, C1/2, C3/4, C5/6, T7/8, CP1/2, CP3/4, CP5/6, TP7/8, TP9/10, P1/2, P3/4, P5/6, P7/8, PO3/4, PO7/8, O1/2). Electrode AFz served as the ground electrode and FCz as the online reference. The recordings were then re-referenced offline to the average of near-mastoid electrodes (TP7/8). Electrodes FP1/2, located above the eye-brows, were used to monitor blinks. Electrode IO was placed on the outer canthus of the right eye to capture horizontal eye movements. Electrode impedances were kept below 10 kΩ for all electrodes. The recordings were amplified by a BrainAmp MR Plus amplifier (Brain Products, GmbH, Germany) with a bandpass filter of 0.016–200 Hz, and digitized at a sampling rate of 1 kHz.

We analyzed the EEG data with the Brain Vision Analyzer 2.0 software (Brain Products, GmbH, Germany). After re-referencing the EEG, it was segmented into epochs relative to the critical verb. Epochs started 300 ms before the critical verb (i.e., the pre-stimulus baseline) and ended 1200 ms post-onset. Trials with blinks, horizontal eye movements, excessive alpha waves, or excessive muscle movement were manually rejected before analysis (based on visual inspection). We also discarded trials associated with incorrect responses in the GJT. This resulted in approximately 10% of data loss. After cleaning the data, the mean number of trials per condition ranged between 33 and 37 out of 40 (Condition 1: 37; Condition 2: 33; Condition 3: 36; Condition 4: 36), and this difference was significant, *F*(2.01,54.31) = 11.049, *p* < 0.01. Follow-up tests showed that the number of artifact-free trials in Condition 2 was lower than in all other conditions [Condition 2 vs. Condition 1: *F*(1,27) = 18.973, *p* < 0.001, *q^∗^* = 0.008; Condition 2 vs. Condition 3: *F*(1,27) = 14.415, *p* = 0.001, *q^∗^* = 0.017; Condition 2 vs. Condition 4: *F*(1,27) = 11.758, *p* < 0.01, *q^∗^* = 0.025], which did not differ from one another. Although this is not ideal, it should not be problematic for mean amplitude analyses (as opposed to peak analyses, which we did not conduct). As explained by Luck (2014, supplement, chapter 8, pp. 4–5), when measuring mean amplitudes, different numbers of trials per condition will not yield a spurious effect and should not be considered a confound. Following artifact rejection, data were baseline-corrected relative to the pre-stimulus baseline and averaged per condition and per subject. Finally, we applied a 30-Hz low-pass filter to the waveforms.

Event-related potentials were then quantified as mean amplitudes in two time windows: 250–450 ms, which corresponds to the LAN/N400, and 500–1000 ms, which corresponds to the P600. Both time-windows are consistent with previous reports on agreement. Importantly, they are the same time windows that we examined in Alemán Bañón and Rothman (2016). Thus, both time windows are the best estimates of where effects of agreement/markedness should emerge. For statistical analysis, we also used the same nine regions of interest (ROI) as in Alemán Bañón and Rothman (2016). Each ROI was calculated by averaging across the mean amplitudes of all electrodes in the region (left anterior: F1, F3, F5, FC1, FC3, FC5; right anterior: F2, F4, F6, FC2, FC4, FC6; left medial: C1, C3, C5, CP1, CP3, CP5; right medial: C2, C4, C6, CP2, CP4, CP6; left posterior: P1, P3, P5, P7, PO3, PO7; right posterior: P2, P4, P6, P8, PO4, PO8; midline anterior: Fz, FCz; midline medial: Cz, CPz; midline posterior: Pz, POz). The resulting values were then submitted to a repeated-measures ANOVA with Markedness (first-person singular subject, third-person singular subject), Agreement (grammatical, ungrammatical), Anterior–Posterior (anterior, medial, posterior), and Hemisphere (left, right) as the repeated factors. Since the hemisphere and midline regions comprise different numbers of electrodes, they were analyzed separately. For the analyses on the midline regions, Anterior–Posterior was the only topographical factor in the model. The Geisser and Greenhouse correction was applied in cases where sphericity could not be assumed. In such cases, we report corrected degrees of freedom (Field, 2005). A false discovery rate correction (Benjamini and Hochberg, 1995) was applied to all follow-up tests, to avoid an inflated Type I error. For all follow-up tests, we provide both the raw *p*-value and the adjusted significance level (*q^∗^*), that is, the significance level below which we consider effects significant.

## Results

All relevant data are provided in Supplementary File 2.

### Behavioral

Table 2 provides the percentage of accurate responses in the GJT for each of the four experimental conditions (together with standard deviations). D-prime scores are also provided in the rightmost column. As can be seen, accuracy was generally very high (above 90% across the board), although participants were less accurate rejecting “unmarked subject violations.” A repeated-measures ANOVA with Markedness (first-person, third-person singular subject) and Agreement (grammatical, ungrammatical) as the repeated factors revealed a main effect of Markedness, *F*(1,27) = 9.051, *p* < 0.01, a main effect of Agreement, *F*(1,27) = 10.731, *p* < 0.01, and a Markedness by Agreement interaction, *F*(1,27) = 10.662, *p* < 0.01. Follow-up tests to the interaction revealed that the main effect of Agreement was only significant in the conditions with third-person singular subjects, *F*(1,27) = 13.316, *p* = 0.001, *q^∗^* = 0.025, driven by the fact that participants were less accurate rejecting ungrammatical sentences than accepting grammatical ones.

**Table 2:** Mean accuracy rates in the Grammaticality Judgment Task for the conditions examining person agreement with first-person singular subjects (i.e., marked subjects) vs. third-person singular subjects (i.e., unmarked subjects) (*N* = 28).

	Grammatical	Violation	D-prime score
Marked-subject	98 (2)	98 (4)	4.1 (0.4)
Unmarked-subject	98 (2)	92 (9)	3.6 (0.7)


*Standard deviations are provided between parentheses.*

### ERP Effects

**FIGURE 1:**
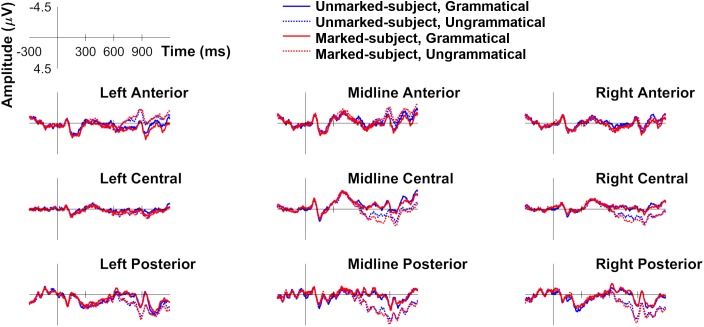
Grand average ERP waveforms for the conditions examining person agreement with unmarked (third person) and marked (first person) subjects: unmarked-subject grammatical, unmarked-subject ungrammatical, marked-subject grammatical, marked-subject ungrammatical.

**FIGURE 2:**
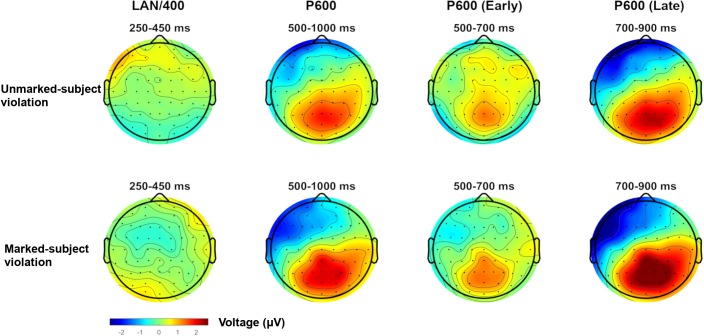
Topographic plots for the two types of person violations (unmarked-subject violation, marked-subject violation) in the 250–450, 500–1000, 500–700, and 700–900 ms time windows. Plots were computed by subtracting the grammatical sentence from the violation condition.

Figure 1 plots ERPs for all four experimental conditions in the six ROIs computed for analysis. As can be seen, approximately 500 ms after presentation of the critical verb, both types of person violations yielded a positivity relative to their grammatical counterparts. In both cases, the positivity shows a central-posterior distribution and a slight right hemisphere bias, consistent with the P600 (e.g., Barber and Carreiras, 2005). In addition, the positivity does not go back to baseline before the end of the epoch (at 1200 ms). The positivity appears more robust for “marked subject violations,” as it almost completely engulfs the positivity for the opposite error type, especially between 700 and 900 ms. This is also visible in Figure 2, which plots the magnitude of the violation effects for both types of person dependencies in four time windows of interest.

Also at approximately 700 ms, both types of person violations become more negative than grammatical sentences in the left anterior region, an effect that also remains visible until the end of the epoch (see Figure 1, 2). This late left anterior negativity also appears larger for “marked subject violations.” Preceding the P600, no evidence for a LAN or an N400 is apparent in Figure 1 or 2 for either type of person violation (e.g., Nevins et al., 2007; Silva-Pereyra and Carreiras, 2007). The following statistical analyses were conducted in the 250–450 ms time window (i.e., LAN effects should emerge in left anterior; N400 effects should emerge primarily in central-parietal regions) and the 500–1000 ms time window (i.e., P600 effects should emerge in central-posterior regions, possibly spanning over frontal regions for “marked subject violations”).

#### Time Window Between 250 and 450 ms (LAN/N400)

Results of the omnibus ANOVA for the 250–450 ms time window are provided in Table 3. As can be seen, the ANOVA revealed two relevant interactions, Agreement by Hemisphere by Anterior–Posterior and Markedness by Agreement by Anterior–Posterior. To follow up on the former, we examined the main effect of Agreement within each of the six relevant ROIs, but no significant effects emerged. To evaluate the second interaction, we examined the Markedness by Agreement interaction, which is directly relevant to our discussion, at each level of Anterior–Posterior. The Markedness by Agreement interaction was significant in the Anterior and Posterior regions, but only before correcting for Type I error [posterior: *F*(1,27) = 6.435, *p* = 0.0172, *q^∗^* = 0.017; anterior: *F*(1,27) = 4.491, *p* = 0.043, *q^∗^* = 0.033]. In the posterior area, the interaction was driven by the fact that “unmarked subject violations” tended to be more negative than grammatical sentences, possibly signaling an N400 effect. However, this effect, which is too small to be visible in the waveforms, was only marginal, even before correcting for Type I error, *F*(1,27) = 3.149, *p* = 0.087, *q^∗^* = 0.008. In contrast, “marked subject violations” tended to be more positive than their grammatical counterparts (possibly signaling the onset of the P600), a comparison that also failed to reach significance. In the anterior area, the interaction was driven by the fact that “unmarked subject violations” were more positive than their grammatical counterparts, while the opposite error type yielded more negative waveforms than correct sentences. None of these comparisons reached significance either.

**Table 3:** Results of the omnibus ANOVA in the 250–450 and 500–1000 ms time windows.

	250–450 ms	500–1000 ms
**Lateral regions: Effects**		
Markedness × Agreement × Anterior × Hemisphere	*F*(1.31,35.47) = 2.553	*F*(1.56,42) = 0.03
Agreement × Anterior × Hemisphere	*F*(1.31,35.5) = 4.212^∗^	*F*(1.34,36.07) = 4.68^∗^
Markedness × Anterior × Hemisphere	*F*(2,54) = 1.016	*F*(2,54) = 0.294
Markedness × Agreement × Hemisphere	*F*(1,27) = 0.016	*F*(1,27) = 2.491
Agreement × Hemisphere	*F*(1,27) = 0.351	*F*(1,27) = 15.028^∗∗∗^
Markedness × Hemisphere	*F*(1,27) = 1.244	*F*(1,27) = 0.729
Markedness × Agreement × Anterior	*F*(1.26,34.13) = 6.53^∗∗^	*F*(1.42,38.31) = 5.405^∗^
Agreement × Anterior	*F*(1.19,32.12) = 0.033	*F*(1.12,30.25) = 24.035^∗∗∗^
Markedness × Anterior	*F*(1.4,37.75) = 1.183	*F*(1.22,33) = 0.638
Markedness × Agreement	*F*(1,27) = 0.003	*F*(1,27) = 0.102
Agreement	*F*(1,27) = 0.013	*F*(1,27) = 13.75^∗∗∗^
Markedness	*F*(1,27) = 0.399	*F*(1,27) = 0.436
**Midline regions: Effects**		
Markedness × Agreement × Anterior	*F*(1.42,38.44) = 3.434^∗^	*F*(2,54) = 3.153ˆ
Agreement × Anterior	*F*(1.33,36.03) = 0.452	*F*(1.33,35.9) = 38.588^∗∗∗^
Markedness × Anterior	*F*(2,54) = 0.9	*F*(2,54) = 1.142
Markedness × Agreement	*F*(1,27) = 0.09	*F*(1,27) = 0.143
Agreement	*F*(1,27) = 0.071	*F*(1,27) = 18.414^∗∗∗^
Markedness	*F*(1,27) = 1.35	*F*(1,27) = 0.36


*^∗^*p* ≤ 0.05; ^∗∗^*p* ≤ 0.01; ^∗∗∗^*p* ≤ 0.001; ˆ*p* ≤ 0.1. Where applicable, degrees of freedom were adjusted using the Greenhouse-Geisser correction.*

As shown in Table 3, the omnibus ANOVA revealed that the Markedness by Agreement by Anterior–Posterior interaction was also significant in the midline. Follow-up tests to this interaction yielded a similar pattern of effects to the hemispheres. That is, the Markedness by Agreement interaction was marginal in midline anterior, but only before correcting for Type I error, *F*(1,27) = 4.035, *p* = 0.055, *q^∗^* = 0.017. This interaction was driven by the fact that “unmarked subject violations” were more positive than grammatical sentences, while “marked subject violations” yielded a negativity relative to grammatical sentences. Only the negativity found for “marked subject violations” was significant, but only before adjusting the *p*-values, *F*(1,27) = 5.069, *p* = 0.033, *q^∗^* = 0.008. Visual inspection of the waveforms shows that this is the beginning of the late anterior negativity, which becomes robust in the subsequent time window.

To summarize, our analyses in the 250–450 ms time window revealed no reliable LAN or N400 effects for either type of person violation, as is clear from Figure 2 (250–450 ms time window). What we see is a trend toward an earlier onset of the late anterior negativity for “marked subject violations.” Additional analyses were conducted in the 300–500 ms time window (e.g., Silva-Pereyra and Carreiras, 2007; Mancini et al., 2011a), which revealed a similar pattern. Thus, we do not report them here.

#### Time Window Between 500 and 1000 ms (P600)

Table 3 summarizes the results of the omnibus ANOVA in the 500–1000 ms time window. As can be seen, the ANOVA revealed a main effect of Agreement, which was qualified by an interaction with Hemisphere and an interaction with Anterior–Posterior. In addition, the Agreement by Hemisphere by Anterior–Posterior interaction was significant. To follow up on the three-way interaction, we first examined the main effect of Agreement within each of the six relevant ROIs. The main effect of Agreement was significant in right posterior, *F*(1,27) = 47.476, *p* < 0.001, *q^∗^* = 0.006; left posterior, *F*(1,27) = 29.587, *p* < 0.001, *q^∗^* = 0.012; and right medial, *F*(1,27) = 22.144, *p* < 0.001, *q^∗^* = 0.019. In addition, it was marginal in left medial before correcting for Type I error, *F*(1,27) = 3.748, *p* = 0.063, *q^∗^* = 0.037. In all cases, person violations overall yielded more positive waveforms than grammatical sentences, consistent with the P600. The main effect of Agreement was also significant in left anterior, *F*(1,27) = 16.206, *p* < 0.001, *q^∗^* = 0.025, but here violations yielded more negative waveforms than grammatical sentences.

At least two factors seem to contribute to this three-way interaction. First, the positivity appears larger in the right hemisphere, as Figure 1, 2 clearly show. This was confirmed by the fact that, when comparing the main effect of Agreement in right posterior and left posterior, the Agreement by Hemisphere interaction was significant, *F*(1,27) = 8.54, *p* < 0.01, *q^∗^* = 0.031, and driven by the positivity being larger in right posterior. However, when comparing the main effect of Agreement in right posterior and right medial, the Agreement by Anterior–Posterior interaction was not significant. The second factor that seems to contribute to the interaction is the fact that an effect of different polarity (i.e., a negativity) emerged for violations in left anterior.

The omnibus ANOVA also revealed a significant Markedness by Agreement by Anterior–Posterior interaction (see Table 3). Since Markedness and Agreement are the two relevant linguistic factors in our study, we followed up on this interaction by examining the Markedness by Agreement interaction at each level of Anterior–Posterior. The interaction was only significant in the anterior portion of the scalp, *F*(1,27) = 6.568, *p* = 0.016, *q^∗^* = 0.02, driven by the fact that “marked subject violations” were more negative than their grammatical counterparts, *F*(1,27) = 9.581, *p* = 0.005, *q^∗^* = 0.01. However, no effects emerged for the opposite type of person error. The larger late left anterior negativity for “marked subject violations” is clearly visible in Figure 2 (500–1000 ms time window).

In the midline, the effects were qualitatively similar to the hemispheres (see Table 3). The Markedness by Agreement by Anterior–Posterior interaction was marginal (*p* = 0.051), and it was driven by the fact that Markedness and Agreement only interacted in midline anterior, but only before correcting for Type I error, *F*(1,27) = 3.44, *p* = 0.075, *q^∗^* = 0.017. Similar to the hemispheres, this interaction was driven by the fact that “marked subject violations” were more negative than grammatical sentences (before adjusting the *p*-values), *F*(1,27) = 6.533, *p* = 0.017, *q^∗^* = 0.008, while the reverse error type yielded no effects.

Finally, follow-up tests to the Agreement by Anterior–Posterior interaction (see Table 3) revealed main effects of Agreement in midline posterior, *F*(1,27) = 50.31, *p* < 0.001, *q^∗^* = 0.017, and midline medial, *F*(1,27) = 23.059, *p* < 0.001, *q^∗^* = 0.033, driven by person violations being more positive than grammatical sentences.

To summarize, our analyses in the 500–1000 ms time window revealed robust P600 effects for both types of person violations in central-posterior areas of the scalp, with a slight right-hemisphere bias. The larger P600 effect that can be seen for “marked subject violations” relative to the reverse error type was, however, not statistically supported in this time window. In the same time window as the P600, person violations also showed an anterior negativity, mainly in left anterior but also present in midline anterior. This negativity is driven by “marked subject violations,” as confirmed by the Markedness by Agreement interaction.

#### Time Window Between 700 and 900 ms (Late Phase of the P600)

To further explore the P600 magnitude difference between the two types of person violations, we conducted additional analyses in the 700–900 ms time window, corresponding to the late phase of the P600 (e.g., Barber and Carreiras, 2005; Silva-Pereyra and Carreiras, 2007). This is when both types of person violations seem to differ the most, as can be seen in Figure 1, 2. We created an additional ROI including the electrodes from all four regions where the P600 was significant: right medial, right posterior, midline medial, and midline posterior. This approach allows us to compare the two types of person violations in all ROIs where we know the P600 emerged, without directly comparing regions with different numbers of electrodes (hemisphere regions: six electrodes; midline regions: two electrodes). A repeated-measures ANOVA with Markedness and Agreement as the repeated factors revealed a significant main effect of Agreement, *F*(1,27) = 48.455, *p* < 0.001, and a significant Markedness by Agreement interaction, *F*(1,27) = 4.508, *p* < 0.05. The interaction was driven by the fact that “marked subject violations” yielded a larger positivity (relative to their grammatical counterparts) than the reverse type of person error.

Additional analyses were conducted in the 500–700 ms time window, which confirmed that the larger P600 for “marked subject violations” was restricted to the 700–900 ms time window (see the topographical plot for the 500–700 ms time window in Figure 2). These analyses only revealed a significant main effect of Agreement, *F*(1,27) = 12.478, *p* < 0.01.

## Discussion

The present study used ERP to investigate subject–verb person agreement in Spanish, with a focus on how markedness differences with respect to the speech participant status of the subject influence agreement resolution at the verb. We manipulated markedness by probing both third-person singular lexical subjects, such as *la viuda* “the widow,” and subjects consisting of the first-person singular pronoun *yo* “I.” Crucially, while first person is marked (i.e., it plays the *speaker* role in the speech act), third person functions as a default, since it plays neither the *speaker* nor the *addressee* role. Our design also manipulated agreement, by crossing third-person singular subjects with first-person singular verbs and vice versa. We hypothesized that person violations might yield an earlier and larger P600 when realized on a marked verb (*la viuda…^∗^lloro* “the widow-_3RD PERSON-SG_ cry-_1ST PERSON-SG_”) relative to an unmarked one (*yo…^∗^llora* “I-_1ST PERSON-SG_ cry-_3RD PERSON-SG_”). This is because violations have been argued to be more disruptive when they are realized on marked items (e.g., Deutsch and Bentin, 2001; Kaan, 2002; Nevins et al., 2007). In addition, this would be in line with what we found for noun–adjective number and gender agreement in Spanish with the same participants (Alemán Bañón and Rothman, 2016). Alternatively, we evaluated the possibility that the marked status of the first-person subject would allow the parser to generate a stronger prediction regarding the upcoming verb due to feature activation (e.g., Nevins et al., 2007). If such is the case, we predicted that violations with a first-person singular subject (*yo…^∗^llora* “I-_1ST PERSON-SG_ cry-_3RD PERSON-SG_”) would show a larger (or more broadly distributed) P600 than violations with unmarked subjects (*la viuda…^∗^lloro* “the widow-_3RD PERSON-SG_ cry-_1STPERSON-SG_”).

Our results revealed that both types of person violations elicited a robust positivity relative to grammatical sentences between 500 and 1000 ms, consistent with the P600, a component that is sensitive to a number of morphosyntactic operations, including agreement (e.g., Hagoort et al., 1993; Osterhout and Mobley, 1995; Nevins et al., 2007; Mancini et al., 2011a). Subsequent analyses revealed that this effect was larger for “marked subject violations,” relative to the opposite error type between 700 and 900 ms. Our results did not reveal any reliable negativities preceding the P600 for either type of person violation (e.g., Nevins et al., 2007; Silva-Pereyra and Carreiras, 2007; cf. Mancini et al., 2011a, 2018; Zawiszewski et al., 2016). However, an anterior negativity did emerge in the P600 time window [similar to Alemán Bañón and Rothman’s study (2016) for both number and gender errors], which was also impacted by markedness, as it was larger for “marked subject violations.” We discuss these effects below.

### Effects of Agreement

The P600 effects for both types of person violations are consistent with a large literature on agreement processing (e.g., Osterhout and Mobley, 1995; Barber and Carreiras, 2005; Alemán Bañón et al., 2012), including all previous studies on person agreement (Rossi et al., 2005; Nevins et al., 2007; Silva-Pereyra and Carreiras, 2007; Mancini et al., 2011a, 2018; Zawiszewski et al., 2016). As previously discussed, the functional significance of the P600 is still a matter of debate. Initial proposals viewed the P600 as an index of syntactic reanalysis and repair (or syntactic difficulty, more generally) (e.g., Osterhout and Holcomb, 1992; Hagoort et al., 1993). Subsequent ones have posited that the P600 reflects reanalysis processes in general (i.e., not exclusively morphosyntactic) (e.g., Kuperberg, 2007; Bornkessel-Schlesewsky and Schlesewsky, 2008), or conflict monitoring (van de Meerendonk et al., 2010). Our results do not adjudicate between these proposals (nor was it the purpose of the study), but they are consistent with them. That is, the P600 effects for person violations here might reflect the reprocessing costs associated with trying to reconcile conflicting information (i.e., morphosyntactic and discourse information) in light of top-down expectations.

The lack of an N400 effect for both types of person violations deserves some discussion. An N400 effect was reported by Mancini et al. (2011a) for person violations in Spanish, and for person errors in Basque by both Zawiszewski et al. (2016) and Mancini et al. (2018). Mancini et al. (2011a) interpret this effect as evidence that person violations disrupt the assignment of a discourse role to the subject, due to the failure to map morphosyntactic and discourse information (i.e., person inflection on the verb + speech participant role). We agree that this is indeed possible, but we remain skeptical about how generalizable this account is, since our results did not reveal N400 effects for either type of person error (consistent with Nevins et al., 2007 and Silva-Pereyra and Carreiras, 2007).

Finally, person violations in the present study also elicited a late anterior negativity in the same time window where the P600 emerged. This effect has been reported in previous studies on agreement that required participants to provide a sentence-final judgment (e.g., Sabourin and Stowe, 2004; Gillon-Dowens et al., 2010; Alemán Bañón et al., 2012; Alemán Bañón and Rothman, 2016; Zawiszewski et al., 2016). One position in the literature is that this late negativity reflects the cost of keeping the ungrammaticalities in working memory until the end of the sentence. This interpretation is consistent with our results. It also explains why the negativity was less robust for “unmarked subject violations” relative to the opposite error type, as participants were less accurate rejecting the former in the GJT (92 vs. 98% accuracy, respectively). One possibility is that the parser can better maintain the feature specification of the subject in the focus of attention when the subject is marked, which would explain why our participants were more accurate rejecting “marked subject violations” in the GJT (e.g., Wagers and McElree, unpublished). Another possibility is that, Spanish being a null-subject language, the salience of an overt personal pronoun facilitated the detection of the ungrammaticalities at the verb. We come back to this possibility below.

In Alemán Bañón and Rothman (2016), we hinted that this late anterior negativity might be a phase reversal of the P600 (Nunez and Srinivasan, 2006), since both effects showed similar latency, but the reverse scalp distribution (right posterior vs. left anterior). The same is true of the late anterior negativity in the present study (see Figure 2). That both components were impacted by markedness in a similar way makes us wonder the extent to which these two components are independent from one another (although Osterhout and Hagoort, 1999 point out that two different ERPs can be impacted by the same factor). We, therefore, remain cautious in interpreting this effect.

### Effects of Markedness

Our results revealed that person agreement violations realized at the verb yielded P600 effects of different magnitude in the 700–900 ms time window, depending on the speech participant status of the subject. More specifically, violations with a first-person singular subject, which corresponds to the *speaker* role, yielded a larger positivity than errors with a third-person (lexical) subject, which is underspecified for person (e.g., Harley and Ritter, 2002). This pattern of results is consistent with the proposal that, upon encountering a subject with marked features, feature activation allows the parser to generate a stronger prediction regarding the upcoming verb (e.g., Nevins et al., 2007). We do not argue that the larger P600 reflects prediction disconfirmation itself, since the effect was not frontally distributed (e.g., DeLong et al., 2011; Van Petten and Luka, 2012) (see Figure 2). The larger P600 for person violations with a marked subject might index the reanalysis process that the parser initiates when there is a conflict between a highly expected verbal form (i.e., more so than in the conditions with an unmarked subject) and the form that was actually encountered (e.g., van de Meerendonk et al., 2010).

These results are not consistent with our previous investigation on the role of markedness in the processing of noun–adjective number and gender agreement in Spanish, which involved the same participants (Alemán Bañón and Rothman, 2016). In that study, we found that violations realized on marked adjectives (plural for number; feminine for gender) yielded earlier and, in the case of number, larger P600 effects than violations realized on unmarked adjectives. Here, we found the reverse. It is possible that differences between the target structures where we examined agreement in each study explain this discrepancy. As we discussed above, the configuration where we examined noun–adjective agreement (e.g., *una catedral que parecía inmensa* “a cathedral-_FEM-SG_ that looked huge-_FEM-SG_”) might not have been sufficiently constraining to allow the parser to generate strong predictions regarding upcoming adjectives, since other continuations were possible (e.g., *una catedral que parecía desafiar la gravedad* “a cathedral that seemed to defy gravity”). In fact, adjective phrases are always optional, although some structures might make adjectives more predictable (e.g., *una fruta muy jugosa* “a fruit-_FEM-SG_ very juicy-_FEM-SG_,” where the adverb *muy* “very” makes it very likely that an adjective will follow; see Alemán Bañón et al., 2012). The same is not true of subject–verb agreement, where the presence of a subject DP allows for the strong prediction that a verb phrase (VP), headed by a verb, will appear in order to satisfy the phrase structure rule for sentence building (e.g., Chomsky, 1957, 1995). It is, therefore, possible that markedness influences agreement processing in different ways at different stages, depending on the nature of the computation itself (see Dillon et al., 2013, who suggested agreement attraction in comprehension to be sensitive to the predictability of the dependency).

The results of the present study differ from those by Mancini et al. (2018) in a number of ways, although there are certain similarities. Unlike Mancini et al. (2018), our results did not reveal reliable N400 effects for either type of person violation, although this is consistent with previous studies (e.g., Nevins et al., 2007; Silva-Pereyra and Carreiras, 2007). With respect to the P600, the present study found that “marked subject violations” yielded a larger P600 than the reverse configuration. A similar asymmetry between violations with marked vs. unmarked plural subjects emerged in Mancini et al.’s study (2018), except that, in their study, only violations with first-person plural subjects yielded a P600. Recall, however, that the ungrammatical status of “third-person plural subject + first-person plural verb” errors in Mancini et al.’s study (2018) was uncertain, given that participants accepted them at a rate of 42% in the judgment task, consistent with theoretical accounts of person agreement in Basque (e.g., Torrego and Laka, 2015; see Mancini et al., 2018 for counterarguments). In addition, the authors did not discard incorrectly judged trials from analysis. The same was not true of our study, where “unmarked subject violations” were unambiguously ungrammatical. In fact, our participants only accepted them at a rate of 8% (and we discarded incorrectly judged trials from analysis). This might explain, partly, why a P600 did not emerge for errors with unmarked subjects in Mancini et al.’s study.

Mancini et al. (2018) interpret their results as evidence that, when the subject carries no person specification (i.e., third-person plural), encountering a verb with first-person plural features (i.e., marked for person) allows the parser to extend the verb’s person specification to the subject. The authors point out that such a process only applies to plural subjects, which include more than one entity. For example, first-person plural includes the *speaker* + associates, and second-person plural includes the *addressee* + associates. In contrast, singular subjects are atomic entities that can only take their canonical speech role. What this means is that Mancini et al.’s proposal cannot explain our findings, since we found an asymmetry in the same direction as they did, but for person errors with singular subjects that differed with respect to markedness. However, Mancini et al.’s results can be explained in terms of an interplay between markedness and top-down expectations. That is, it is possible that the marked status of the first/second-person plural suffix –*ok*, relative to the third-person plural suffix –*ek*, allowed the parser to generate a stronger prediction regarding the upcoming verb. Future studies should explore this possibility, for example, by looking at person dependencies with plural subjects in non null-subject languages, where “third-person plural subject + first-person plural verb” configurations are more categorically disallowed (Höhn, 2016)^5^.

We must point out, however, that first- and third-person subjects in our study differed with respect to more than just feature specification. While the first-person conditions involved a personal pronoun, the third-person conditions involved referential DPs, and the reader might rightfully wonder how this could have affected our results. Recall that we opted for lexical DPs (as opposed to third-person pronouns) because there is consensus in the literature that they carry no person specification. Therefore, only first-person subjects should have allowed for prediction generation with respect to person morphology at the verb^6^.

One possibility is that sentences with first-person subjects were more salient than sentences with referential subjects because Spanish licenses *pro* drop and personal pronouns are often null. While this is indeed possible, we point out that overt pronouns are syntactically licensed and pragmatically appropriate as subjects in Spanish. Null pronouns are preferred as subjects if their referent can be inferred from context (topic maintenance), whereas overt pronouns tend to be used when there is a discourse switch to another referent (topic shift) (e.g., Lubbers-Quesada and Blackwell, 2009) or for contrastive focus (e.g., Rothman, 2009). This division of labor clearly emerges in cases of anaphora resolution such as *the man pushed the boy when he/Ø..*. Here, null pronouns have been found to prefer subjects (*the man*) (e.g., Alonso-Ovalle et al., 2002; Carminati, 2005; Filiaci et al., 2014) and overt pronouns, objects (*the boy*) (e.g., Alonso-Ovalle et al., 2002; cf. Filiaci et al., 2014). Our materials, however, did not require anaphoric resolution. In fact, since each sentence was presented with no prior context (one that would determine topic maintenance or shift), the use of an overt pronoun does not seem overtly salient. In addition, we are skeptical that the use of third-person singular pronouns would have ameliorated this issue (even beyond theoretical considerations). Such a strategy would have made third-person pronouns more salient, because of their lower proportion in the language overall. For example, Morales (1997) shows that the proportion of overt first-person singular pronouns in European Spanish (our participants’ variety) is 28%, compared to 8% for third-person pronouns (see similar results in Duarte and Soares da Silva, 2016).

Another possibility is that the parser might have extracted feature information more easily from personal pronouns than lexical DPs, which encode lexical information that can slow down processing. While we cannot rule out this possibility, we point out that the adverb *a menudo* “often” intervened between the subject and the verb. Thus, since we used a 750 ms stimulus onset asynchrony, participants had 1800 ms to extract person information from the subject before encountering the verb (*la viuda a menudo VERB*). This time interval should have allowed participants to generate predictions (e.g., Chow et al., 2018b). Alternatively, the semantic features of lexical DPs might have impacted processing at the verb, either by allowing the parser to predict the type of event encoded by the verb, or by allowing combinatorial processing with the verb’s semantic features (even if the verb itself was not predicted). While this is also possible, we point out that the verb was held constant in the grammatical and ungrammatical conditions (*la viuda…llora/^∗^lloro*). Thus, this should not have impacted the violation effect. We examined the possibility that lexical DPs might have allowed the parser to predict the event described by the verb by calculating the cloze probability of the target verbs in these conditions. The results of this cloze test (*N* = 33) show that mean cloze probability (across items) was very low (mean = 0.03; SD: 0.1), and that only one item had a cloze probability over 0.67, which corresponds to high probability (e.g., Block and Baldwin, 2010). Thus, the target verbs in the conditions with DP subjects were, overall, not predictable. Future research should investigate how markedness modulates person agreement while controlling for these differences, for example, by introducing the two subjects in a previous context, in order to reduce the salience of *yo* in the sentence where agreement is manipulated, and by using demonstratives in lieu of lexical DPs or third-person pronouns (see 9).

**Table:** 

(9)	El atleta	y yo vamos al	gimnasio.
	The athlete	and I go	to-the gym
	a.	Yo	entreno/^∗^entrena…
		I	train-_1ST PERSON-SG_/train-_3RD PERSON-SG_
	b.	Éste	entrena/^∗^entreno…
		This	train-_3RD PERSON-SG_/train-_1ST PERSON-SG_

Two additional issues, however, might seem to undermine our claims. First, the mean number of trials for “unmarked subject violations” (Condition 2) was significantly lower than in the other three conditions. Thus, one could easily argue that the smaller P600 for person errors with an unmarked subject could be accounted for by signal-to-noise ratio differences across the conditions being compared. We can provide two counterarguments, one methodological and one theoretical. First, as discussed above, Luck (2014) points out that differences with respect to the mean number of trials per condition may affect analyses based on peak amplitudes, which we did not conduct, but not comparisons based on mean amplitudes, which are the basis for our conclusions. We therefore assume that the P600 size differences between the two error types are not epiphenomenal. Notice also that, albeit significant, the numerical differences in number of items across conditions were rather small (Condition 1: 37; Condition 2: 33; Condition 3: 36; Condition 4: 36) and the mean number of good items per condition was well above 30 across the board. Our second argument is that we only retained for analysis artifact-free trials that the participants had correctly judged in the GJT (unlike Mancini et al., 2018). As discussed in Section “Results,” participants were least accurate rejecting “unmarked subject violations” (Condition 2). Thus, the fact that Condition 2 encompassed fewer trials than the other conditions is not independent from how markedness impacts person agreement resolution online, which is our main research question.

The second issue concerns differences in lexical frequency between the critical verbs. Recall that first-person verbs were significantly less frequent than third-person ones. How could this have affected our results? There is evidence in the literature that lexical frequency is inversely related to the amplitude of the N400 (e.g., Neville et al., 1992; Kutas et al., 2006), a component associated with lexical access and retrieval. That is, less frequent words tend to show a larger N400. One possibility is that violations realized on first-person verbs (*la viuda…^∗^lloro* “the widow-_3RD PERSON-SG_ cry-_1ST PERSON-SG_”) yielded more negative effects than their grammatical counterparts (*la viuda…llora* “the widow-_3RD PERSON-SG_ cry-_3RD PERSON-SG_”) in the N400 time-window, due to the fact that the verb was less frequent in the violation condition. In turn, this might have attenuated the following P600. Moreover, the reverse could have happened in the conditions with a marked subject. That is, violations on third-person singular verbs (*yo…^∗^llora* “I-_1ST PERSON-SG_ cry-_3RD PERSON-SG_”) might have elicited a smaller N400 relative to their grammatical counterparts (*yo…lloro* “I-_1ST PERSON-SG_ cry-_1ST PERSON-SG_”), due to the fact that the verb was more frequent in the ungrammatical condition. In turn, this might have amplified the size of the subsequent P600. In fact, the results reported for the N400 time window are compatible with this scenario. Those analyses revealed a trend toward an N400 for “unmarked subject violations,” and a trend toward a positivity for “marked subject violations.” Crucially, however, the effects of markedness in our study emerged between 700 and 900 ms, in the late phase of the P600. If differences in the N400 time window (caused by differences in lexical frequency between the critical verbs) were responsible for the difference in P600 size across markedness conditions, those differences should have been largest in the early phase of the P600, right after the N400 (500–700 ms), which was not the case. To rule out this possibility, we recalculated effects in the 700–900 ms time window by using the N400 time window as a baseline (we used both the 250–450 and the 300–500 ms time windows) (e.g., Hagoort, 2003; Wicha et al., 2004; Martín-Loeches et al., 2006). These analyses revealed a similar pattern of results as with a pre-stimulus baseline. That is, the P600 was larger for marked subject violations, relative to violations with a third-person subject^7^. Thus, we can safely assume that the markedness effects that we found in the P600 time window are, at least to some extent, independent of baseline differences.

## Conclusion

The data reported in the present study showed that subject–verb person agreement resolution in Spanish is impacted by the speech participant status of the subject. More specifically, we found that person violations where the subject is the *speaker* (i.e., first person, marked for person) yielded a larger P600 between 700 and 900 ms than violations where the subject is not a speech participant (i.e., third person, the default person). We interpreted these findings as evidence that, upon encountering a marked element (i.e., the subject), feature activation allows the parser to generate a stronger prediction regarding the form of the upcoming verb (e.g., Nevins et al., 2007). When this prediction is not met, the result is a larger P600 relative to cases when no feature information is available at the subject.

## Author Contributions

JA conceptualized the study, designed the materials, collected the data with help from research assistants, conducted the analyses, and wrote the original draft. JR supervised the study, and contributed to the original draft.

## Conflict of Interest Statement

The authors declare that the research was conducted in the absence of any commercial or financial relationships that could be construed as a potential conflict of interest.

## Footnotes

1 See Harley and Ritter (2002) for further discussion. See Forchheimer (1953) for a more elaborate list of differences between first/second and third person.

2 The Spanish plural personal pronouns *nosotros/nosotras* “we-_MASC/FEM_” and *vosotros/vosotras* “you-_PL-MASC/FEM_” might seem to contradict this observation. However, as Harley and Ritter (2002) point out, these are bimorphemic pronouns, where the actual person morphemes (*nos, vos*) show no gender distinction. They only show person and number specification. Likewise, the morpheme *otros/otras* shows number and gender specification, but not person.

3 Nevins et al. (2007) approached this question by comparing single to double violations, which did not differ from one another, possibly due to the use of subjects with default agreement features. If subject markedness determines, at least to some extent, whether agreement processing is predictive, then differences should emerge when comparing two types of single violations that differ with respect to subject markedness, as in the present study.

4 Mancini et al. (2018) circumvented this issue by looking at auxiliary verbs in Basque.

5 Recall that, in the behavioral literature, Carminati (2005) found that disambiguating verbs inflected for first person carried a smaller reaction-time penalty than verbs inflected for third person. If our proposal is on the right track, it is possible that such an effect arose because of the unmarked nature of the matrix subject, which did not allow for strong predictions regarding upcoming verbs.

6 Lexical DPs might have activated other features (e.g., *la viuda* “the widow” is [+feminine]), but these features were not manipulated at the verb (i.e., the Spanish verb only encodes number, which was held constant, and person). In addition, the use of third-person pronouns would not have mitigated this issue, since they also encode other features, such as gender or animacy (*él/ella/ello* “he/she/it”). In fact, this relates to markedness asymmetries, as we discussed in Section “Introduction.”

7 We provide the relevant results of the analyses using a 300–500 ms baseline. In the midline, we found a Markedness by Agreement interaction, *F*(1,27) = 4.571, *p* < 0.05, driven by “marked subject violations” yielding a larger P600 than the reverse error type. In the hemispheres, we found a marginal Markedness by Agreement by Hemisphere interaction, *F*(1,27) = 3.983, *p* = 0.056, and a marginal Markedness by Agreement by Anterior–Posterior by Hemisphere interaction, *F*(1.32,35.79) = 2.785, *p* = 0.09. Follow-ups showed that the Markedness by Agreement interaction was significant in the right hemisphere, *F*(1,27) = 4.278, *p* < 0.05, driven by the fact that “marked subject violations” yielded a larger P600 than the reverse error type.
