# Supplementary material for: Being a Participant Matters: Event-Related Potentials Show That Markedness Modulates Person Agreement in Spanish
Source: Front Psychol. 2019 Apr 24;10:746. doi: 10.3389/fpsyg.2019.00746 (PMC6491576; doi:10.3389/fpsyg.2019.00746)
Supplement: Supplementary file 1 [file Table_1.DOCX]

**Sentences with a third‑person singular subject**

1. La viuda a menudo llora/*lloro en la iglesia.
2. La azafata a menudo bebe/*bebo en la cafetería.
3. El cazador a menudo acampa/*acampo en la montaña.
4. La bailarina a menudo practica/*practico en el teatro.
5. El ayudante a menudo responde/*respondo a los emails.
6. La enfermera a menudo colabora/*colaboro en la clínica.
7. El cartero a menudo acaricia/*acaricio a los gatos.
8. El conductor a menudo acelera/*acelero en la carretera.
9. La tenista a menudo compite/*compito en el torneo.
10. La psicóloga a menudo aconseja/*aconsejo a su familia.
11. El actor a menudo actúa/*actúo en la ópera.
12. La farmacéutica a menudo enseña/*enseño en la facultad.
13. El perro a menudo asusta/*asusto a mis primos.
14. El dependiente a menudo contradice/*contradigo a su jefe.
15. La vecina a menudo baila/*bailo en el patio.
16. El piloto a menudo aterriza/*aterrizo en Nueva York.
17. La chica a menudo corre/*corro en el gimnasio.
18. El veterinario a menudo atiende/*atiendo a los animales.
19. El ingeniero a menudo busca/*busco a su equipo.
20. El atleta a menudo calienta/*caliento en la pista.
21. La artista a menudo canta/*canto en los festivales.
22. El padre a menudo arropa/*arropo a sus hijos.
23. La pescadera a menudo chilla/*chillo en su tienda.
24. La cocinera a menudo discute/*discuto en la cocina.
25. La actriz a menudo conduce/*conduzco en las películas.
26. La editora a menudo humilla/*humillo a los novelistas.
27. La arqueóloga a menudo confunde/*confundo a sus empleados.
28. El músico a menudo ignora/*ignoro a sus oyentes.
29. El muchacho a menudo esquía/*esquío en los Alpes.
30. La dentista a menudo contesta/*contesto a sus colegas.
31. La niñera a menudo controla/*controlo a sus sobrinos.
32. El diplomático a menudo coopera/*coopero en las asambleas.
33. La pasajera a menudo duerme/*duermo en el autobús.
34. El hombre a menudo cose/*coso en el colegio.
35. La doctora a menudo inspecciona/*inspecciono a sus pacientes.
36. La monja a menudo reza/*rezo en la capilla.
37. El reportero a menudo cree/*creo a sus informantes.
38. La nadadora a menudo entrena/*entreno en su dormitorio.
39. El abuelo a menudo cuida/*cuido a sus nietos.
40. La profesora a menudo fuma/*fumo en el aula.
41. El escritor a menudo defiende/*defiendo a sus lectores.
42. El maestro a menudo felicita/*felicito a sus estudiantes.
43. La vendedora a menudo confía/*confío en la gente.
44. La sirvienta a menudo empuja/*empujo a sus compañeras.
45. El fotógrafo a menudo miente/*miento en los periódicos.
46. La extranjera a menudo entiende/*entiendo a sus compatriotas.
47. El viajero a menudo entra/*entro en la estación.
48. La limpiadora a menudo escupe/*escupo en el suelo.
49. El taxista a menudo recoge/*recojo a los deportistas.
50. El arquitecto a menudo espía/*espío a sus compradores.
51. La directora a menudo golpea/*golpeo a los aprendices.
52. La madre a menudo idealiza/*idealizo a su hija.
53. La embajadora a menudo viaja/*viajo en su motocicleta.
54. El niño a menudo idolatra/*idolatro a sus amigos.
55. El abogado a menudo teme/*temo a los jueces.
56. El detective a menudo persigue/*persigo a los ladrones.
57. La peluquera a menudo intimida/*intimido a sus clientes.
58. La camarera a menudo vigila/*vigilo a los consumidores.
59. El banquero a menudo invita/*invito a sus socios.
60. El bombero a menudo salva/*salvo a las víctimas.
61. La pianista a menudo medita/*medito en los conciertos.
62. La princesa a menudo sigue/*sigo a la reina.
63. El mecánico a menudo interrumpe/*interrumpo en el trabajo.
64. La violinista a menudo nada/*nado en la piscina.
65. La criada a menudo obedece/*obedezco a su marido.
66. La señora a menudo ataca/*ataco a los pájaros.
67. El chico a menudo perdona/*perdono a sus tíos.
68. El investigador a menudo examina/*examino a sus colaboradores.
69. El médico a menudo reconoce/*reconozco a los niños.
70. El ministro a menudo generaliza/*generalizo en sus discursos.
71. El señor a menudo patina/*patino sobre el hielo.
72. El becario a menudo señala/*señalo a su supervisor.
73. La abuela a menudo regaña/*regaño a su nieta.
74. La secretaria a menudo sonríe/*sonrío en el aeropuerto.
75. La periodista a menudo improvisa/*improviso en sus artículos.
76. El científico a menudo tose/*toso en las conferencias.
77. El profesor a menudo aplaude/*aplaudo en las charlas.
78. La mujer a menudo manipula/*manipulo a su esposo.
79. El soldado a menudo reflexiona/*reflexiono en el cuartel.
80. El marinero a menudo navega/*navego en el Caribe.

**Sentences with a first‑person singular subject**

1. Yo a menudo lloro/*llora en las películas.
2. Yo a menudo bebo/*bebe en los bares.
3. Yo a menudo acampo/*acampa en la costa.
4. Yo a menudo practico/*practica en el conservatorio.
5. Yo a menudo respondo/*responde a las cartas.
6. Yo a menudo colaboro/*colabora en la oficina.
7. Yo a menudo acaricio/*acaricia a los caballos.
8. Yo a menudo acelero/*acelera en la autopista.
9. Yo a menudo compito/*compite en los partidos.
10. Yo a menudo aconsejo/*aconseja a mis primos.
11. Yo a menudo actúo/*actúa en el circo.
12. Yo a menudo enseño/*enseña en un colegio.
13. Yo a menudo asusto/*asusta a los niños.
14. Yo a menudo contradigo/*contradice a los jueces.
15. Yo a menudo bailo/*baila en la facultad.
16. Yo a menudo aterrizo/*aterriza en San Francisco.
17. Yo a menudo corro/*corre en el estadio.
18. Yo a menudo atiendo/*atiende a mis clientes.
19. Yo a menudo busco/*busca a mi jefe.
20. Yo a menudo caliento/*calienta en la piscina.
21. Yo a menudo canto/*canta en la ducha.
22. Yo a menudo arropo/*arropa a mis hermanos.
23. Yo a menudo chillo/*chilla en el mercado.
24. Yo a menudo discuto/*discute en el metro.
25. Yo a menudo conduzco/*conduce en el pueblo.
26. Yo a menudo humillo/*humilla a la policía.
27. Yo a menudo confundo/*confunde a los participantes.
28. Yo a menudo ignoro/*ignora a mi familia.
29. Yo a menudo esquío/*esquía en los Pirineos.
30. Yo a menudo contesto/*contesta a mis lectores.
31. Yo a menudo controlo/*controla a mis colegas.
32. Yo a menudo coopero/*coopera en los congresos.
33. Yo a menudo duermo/*duerme en un sillón.
34. Yo a menudo coso/*cose en mi apartamento.
35. Yo a menudo inspecciono/*inspecciona a los testigos.
36. Yo a menudo rezo/*reza en la iglesia.
37. Yo a menudo creo/*cree a mis oyentes.
38. Yo a menudo entreno/*entrena en el gimnasio.
39. Yo a menudo cuido/*cuida a los enfermos.
40. Yo a menudo fumo/*fuma en mi cuarto.
41. Yo a menudo defiendo/*defiende a mis sobrinos.
42. Yo a menudo felicito/*felicita a mis alumnos.
43. Yo a menudo confío/*confía en mis parientes.
44. Yo a menudo empujo/*empuja a mis amigos.
45. Yo a menudo miento/*miente en la asamblea.
46. Yo a menudo entiendo/*entiende a mis compatriotas.
47. Yo a menudo entro/*entra en las discotecas.
48. Yo a menudo escupo/*escupe en la montaña.
49. Yo a menudo recojo/*recoge a mis nietos.
50. Yo a menudo espío/*espía a mis aprendices.
51. Yo a menudo golpeo/*golpea a los boxeadores.
52. Yo a menudo idealizo/*idealiza a mi amante.
53. Yo a menudo viajo/*viaja en mi auto.
54. Yo a menudo idolatro/*idolatra a los políticos.
55. Yo a menudo temo/*teme a las ratas.
56. Yo a menudo persigo/*persigue a mis hijos.
57. Yo a menudo intimido/*intimida a mis superiores.
58. Yo a menudo invito/*invita a mi jefa.
59. Yo a menudo vigilo/*vigila a las víctimas.
60. Yo a menudo salvo/*salva a las ballenas.
61. Yo a menudo medito/*medita en la capilla.
62. Yo a menudo sigo/*sigue a mi socio.
63. Yo a menudo interrumpo/*interrumpe en la escuela.
64. Yo a menudo nado/*nada en el lago.
65. Yo a menudo obedezco/*obedece a la presidenta.
66. Yo a menudo ataco/*ataca a las ardillas.
67. Yo a menudo perdono/*perdona a mis compañeras.
68. Yo a menudo examino/*examina a mis estudiantes.
69. Yo a menudo reconozco/*reconoce a mis pacientes.
70. Yo a menudo generalizo/*generaliza en mis críticas.
71. Yo a menudo patino/*patina en la pista.
72. Yo a menudo señalo/*señala a los ladrones.
73. Yo a menudo regaño/*regaña a mis empleados.
74. Yo a menudo sonrío/*sonríe en las fotos.
75. Yo a menudo improviso/*improvisa en mis charlas.
76. Yo a menudo toso/*tose en el campo.
77. Yo a menudo aplaudo/*aplaude en el teatro.
78. Yo a menudo manipulo/*manipula a mi novia.
79. Yo a menudo reflexiono/*reflexiona en mi cama.
80. Yo a menudo navego/*navega en el océano.
